# A Guide to the Variability of Flavonoids in *Brassica oleracea*

**DOI:** 10.3390/molecules22020252

**Published:** 2017-02-08

**Authors:** Vera Mageney, Susanne Neugart, Dirk C. Albach

**Affiliations:** 1 Institute of Biology and Environmental Sciences, Carl von Ossietzky University, Oldenburg Carl von Ossietzky Str. 9-11, 26129 Oldenburg, Germany; vera.mageney@uni-oldenburg.de; 2 Leibniz-Institute of Vegetables and Ornamental Crops Grossbeeren/Erfurt e. V., Theodor-Echtermeyer-Weg 1, 14979 Grossbeeren, Germany; neugart@igzev.de

**Keywords:** variation, breeding perspectives, secondary metabolites, flavonol, anthocyanidin

## Abstract

Flavonoids represent a typical secondary metabolite class present in cruciferous vegetables. Their potential as natural antioxidants has raised considerable scientific interest. Impacts on the human body after food consumption as well as their effect as pharmaceutical supplements are therefore under investigation. Their numerous physiological functions make them a promising tool for breeding purposes. General methods for flavonoid analysis are well established, though new compounds are still being identified. However, differences in environmental circumstances of the studies and analytical methods impede comparability of quantification results. To promote future investigations on flavonoids in cruciferous plants we provide a checklist on best-practice in flavonoid research and specific flavonoid derivatives that are valuable targets for further research, choosing a representative species of scientific interest, *Brassica oleracea*.

## 1. Introduction

*Brassica oleracea* comprises several crop varieties of worldwide economic importance, such as kale, broccoli, Brussels sprouts and cauliflower. In 2012 their production in the USA covered about 27% of all the acreage used for vegetable production (165,000 acres in total) [1]. Their high intraspecific variability extends to secondary metabolites produced by *Brassica* plants, among them glucosinolates and flavonoids [2,3]. The latter play an important role in ultraviolet (UV) protection since UV-B responsive flavonoids can reduce the risk of reactive oxygen species (ROS) generation and thereby prevent oxidative damage [4]. Therefore, the impact of flavonoids on the human body after food consumption as well as their effect as pharmaceutical supplements was discussed in several reviews [5,6,7,8]. Particularly relevant are their antioxidative activity and radical scavenging capacity [5]. So far, flavonoids are known to protect against the initiation and progression of atherosclerosis and cardiovascular disease [9]. Single flavonoid accumulation in plants through target-oriented breeding approaches as well as detailed quantification data are therefore not only of economic, but also of medical research interest. Here, we point to typical pitfalls and limitations as well as to provide a best-practice guide to generate reproducible data of high informative value.

## 2. Data Comparability: Telling the Whole Story

**Figure 1:**
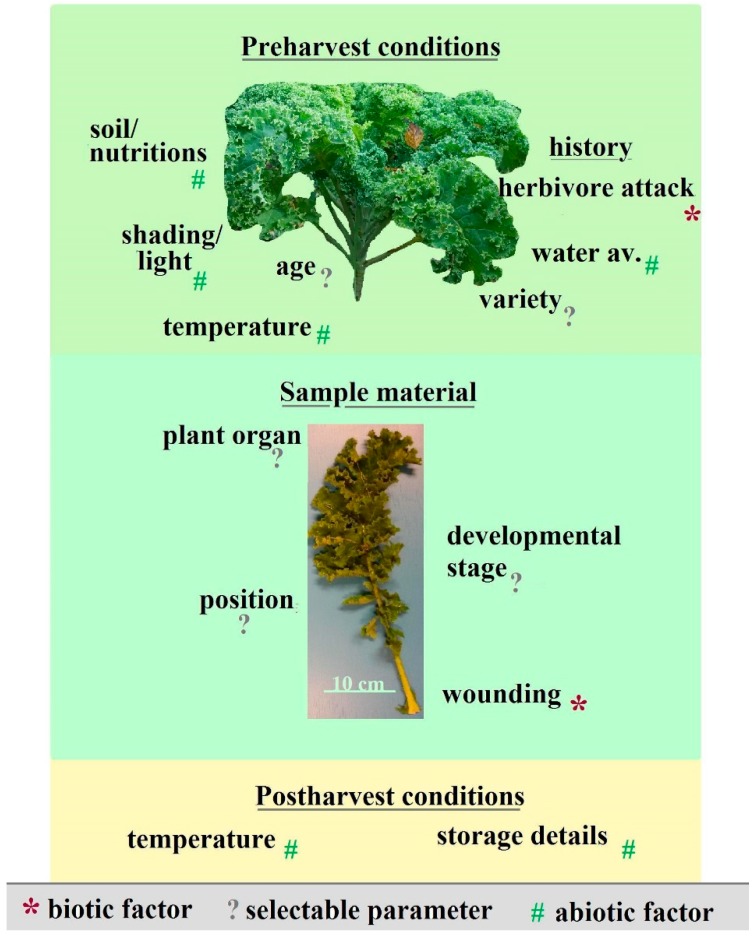
Overview on abiotic, biotic and selectable factors influencing the flavonoid content and composition in plants. Water av.: water availability.

In nature, genotype and ecological parameters influence general phenolic contents (mostly flavonoids and hydroxycinnamic acids) and their antioxidant activity in plant tissue [10]. The necessity to consider seasonal and environmental parameters was demonstrated by numerous studies, for instance Schmidt et al. [11] and Reilly et al. [12], who found strong flavonoid variation with respect to tissue, year and climatic factors in addition to intercultivar variability. However, selectable parameters often remain unmentioned as well, although these specifications are easy to make and likewise necessary for further comparisons. An overview on the complexity of those selectable parameters and influencing factors is given in Figure 1.

Ontogenetic changes in metabolic plant profiles are well documented. Within the first two weeks of plant life, a remarkable decrease is noted regarding phenolic compounds. In Tronchuda cabbage (*B. oleracea* var. *costata*) about 11.1 mg phenols was determined per kg dry weight in sprouts. Ten days later, this value had decreased by 85% [13]. Therefore, phenolic compounds are suggested to play an essential role in early plant development relevant for cell wall biosynthesis and in their function as antioxidants [13]. Later developmental stages in white (*B. oleracea* var. *capitata*) and Chinese cabbage (*Brassica rapa* var. *pekinensis*) are characterized by a significant increase in total flavonoid contents from four weeks after germination to week twelve followed by a gradual decrease [14]. However, it is not clear to what extent ontogenetic or abiotic factors determine this change. As demonstrated by Soengas et al. [15], ontogenetic differences in flavonoid production changes could also be a suitable parameter to distinguish and characterize *B. oleracea* varieties.

Tissue specificity of flavonoid accumulation was analysed in detail quantifying flavonoids in secondary florets, mature primary florets, immature primary florets as well as crop waste parts of three purple cultivars of broccoli [9]. Although great differences between plant organs were found in this study, mean “total flavonoid contents” of all three cultivars were almost alike. Considering leaf material only, Sousa et al. [16] pointed to the importance of distinguishing flavonoid profiles between external and internal leaves based on qualitative differences in flavonoid compositions as well as generally higher phenolic contents of external leaves. In accordance, the same group determined a decrease in antioxidant potential from external leaves to internal leaves of Tronchuda cabbage [17].

Injury caused either by pathogens or herbivores results in catechin and proanthocyanidin accumulation in some species [18], whereas in *Arabidopsis thaliana* damaged leaves show suppressed flavonoid levels [19]. The corresponding authors underline that metabolite movements through the plant initiated by herbivore feeding is often misinterpreted as local accumulation.

Light conditions also affect plants secondary metabolite profile. As demonstrated in broccoli and kale, flavonoid concentration increases with higher photosynthetic active radiation (PAR) levels [20]. Further relevant for light related changes in single flavonoid concentrations is the PAR interaction with temperature [21]. In response to UV-B radiation, qualitative as well as quantitative changes in flavonol compositions were noted [22]. Qualitative differences in flavonol ratios were found in response to low temperature conditions between 0.3 and 9.6 °C as well, whereas no impact of low temperatures on “total flavonoid contents” was supported [11].

The effect of fertilizers on total phenolic content is contradictory discussed in the literature. Comparisons of fertilization with organic matter to conventional fertilizers (nitrogen, boron, and sulphur) imply that organic fertilization induces the acetate/shikimate pathway and therefore lead to higher flavonoid levels, whereas conventional fertilizers result in higher phenolic acid contents [16]. This effect is further supported by a field experiment on broccoli cultivars, which resulted in higher total flavonoid levels in response to organic fertilizer treatment [23]. Based on other data, organic treatment including a four-year rotation system of soil usage, organic fertilisation and winter cover crop did not lead to a significant increase in flavonoid levels [24]. Differences between the mentioned references might be caused by the choice of cultivar, as nutrition responsiveness varies among accessions [25] and distinct flavonoids chosen for quantification. Those comprised primarily catechins and luteolin [24,26], kaempferol glycoside [16] and flavonol quercetin aglycones [23], pointing to the necessity of a standard protocol to enable data comparability.

Regarding post-harvest conditions, the question on how cold storage at 1 °C affects sample material remains unanswered due to contradictory findings supporting flavonoid content preservation over several weeks [27] or pointing to a large decrease of more than 60% within the very first week [28]. Both studies concentrated on flavonol quantifications and therefore do not provide information on flavonoid metabolism in general during postharvest cooling. In contrast to the first study mentioned [27], the latter misses a clear differentiation in this regard [28]. This missing balancing act between single substance quantifications and general statements on flavonoid metabolism are unfortunately common in the literature (see Table 1).

## 3. Dealing with Complexity

**Figure 2:**
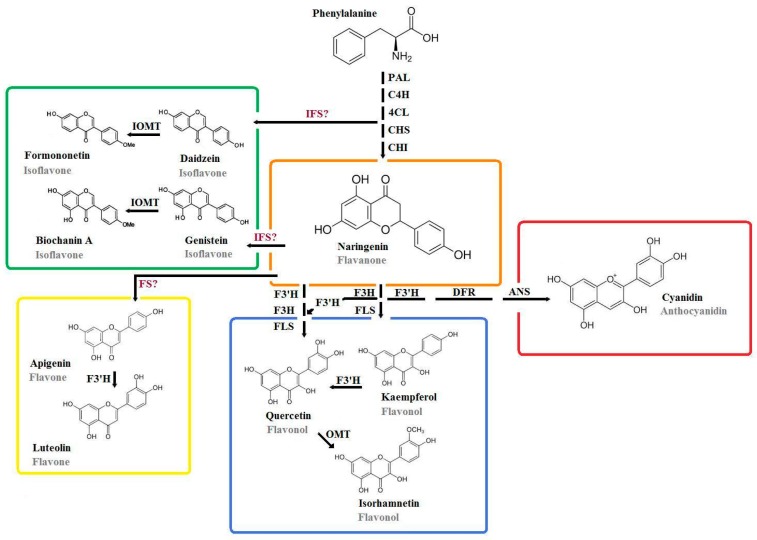
Simplified overview on flavonoid biosynthesis in *Brassica oleracea*. PAL: phenylalanine ammonialyase; C4H: cinnamate-4-hydroxylase; 4CL: 4-coumarate-coenzyme A ligase; CHS: chalcone synthase; CHI: chalcone isomerase; IOMT: isoflavone-*O*-methyltransferase; IFS: isoflavone synthase; FS: flavone synthase; F3H: flavonol-3-hydroxylase; F3’H: flavonol-3’-hydroxylase; FLS: flavonol synthase; DFR: dihydroflavonol reductase; ANS: anthocyanidin synthase; OMT: *O*-methyltransferase.

Flavonoids are phenolic compounds containing an aromatic C_6_ ring bearing at least one hydroxyl group (Figure 2) [34]. Since flavonoids are generally found as glycosides in plant tissues [35] and thus are able to bind different sugar molecules to various positions, one can distinguish about 10,000 forms of flavonoids, and this number continues to increase [4]. For a single aglycone such as quercetin alone, one can find more than 170 different natural glycosides [36].

Due to its high number of glycosidic forms, single flavonoid analyses need to be analysed aglycone- or glycoside-specific. Conflicting results can be caused by the choice of flavonoid glycosides considered in the corresponding studies. Therefore, a more general guideline is required, which gives both quantification parameters as well as potentially valuable derivatives represented in the species of interest. In *B. oleracea*, some flavonoid subclasses are represented in small quantities or not detectable at all, whereas other subclasses have a great potential to provide cultivar- or variety-specific flavonoid profiles.

In contrast to flavanoles and flavanones, numerous representatives of other flavonoid subclasses merit closer consideration based on previous data (Table 2). An additional approach to examine variety specific qualitative and quantitative variability of flavonols was performed by our working group (next section).

## 4. Specific Flavonoids of Major Interest

Depending on their structure, flavonoids are usually separated into six main subclasses [37,38] and subcategorized within it according to their substituents (see Figure 2 for comparison) [39]. Out of these, flavanoles, characteristic of teas, red grapes and red wines, are excluded in this review since their occurrence is not supported for *B. oleracea* [37]. A second group, flavanones such as naringenin, are more relevant as precursors of other flavonoids in *B. oleracea* rather than for their direct accumulation as typical for citrus foods [37].

## 5. Compound Ratios as Plant Character

**Figure 3:**
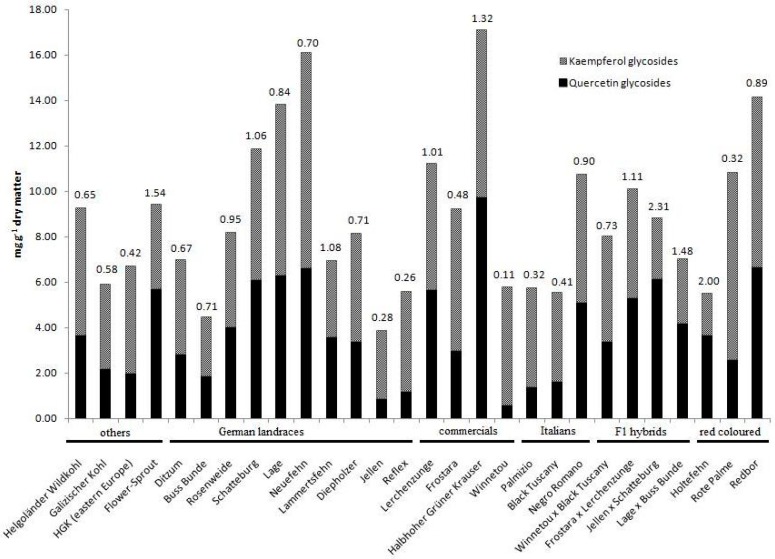
Quercetin and kaempferol glycoside contents and ratios (above bars) quantified in kale cultivars (*Brassica oleracea* convar. *acephala* var. *sabellica*) via HPLC-MS analyses. HGK: “Halbhoher grüner Krauser”. Materials and methods are given in the Appendix.

Our own investigations including 28 cultivars of kale (*B. oleracea* var. *sabellica*) considered main glycosides of the flavonols kaempferol (11 glycosides considered) and quercetin (5 glycosides considered) (see also Supplemental Material). Categorization according to geographical origin or morphological characteristics such as red leaf colour did not provide any significant differences between cultivars. Instead, we found a high variability in single contents and quercetin-to-kaempferol (Q/K) ratios. More precisely, Q/K ratios varied from 0.11 in cultivar “Winnetou” to 2.31 in “Jellen × Schattenburg” (Figure 3). Previous data based on eight kale cultivars reported half of that variation from 0.17 in “Frostara” to 1.02 in “Redbor” [11]. This great variability in Q/K ratios is a promising tool to optimize kale cultivar antioxidant activity inasmuch as quercetin provides higher activity than kaempferol [51,52].

**Figure 4:**
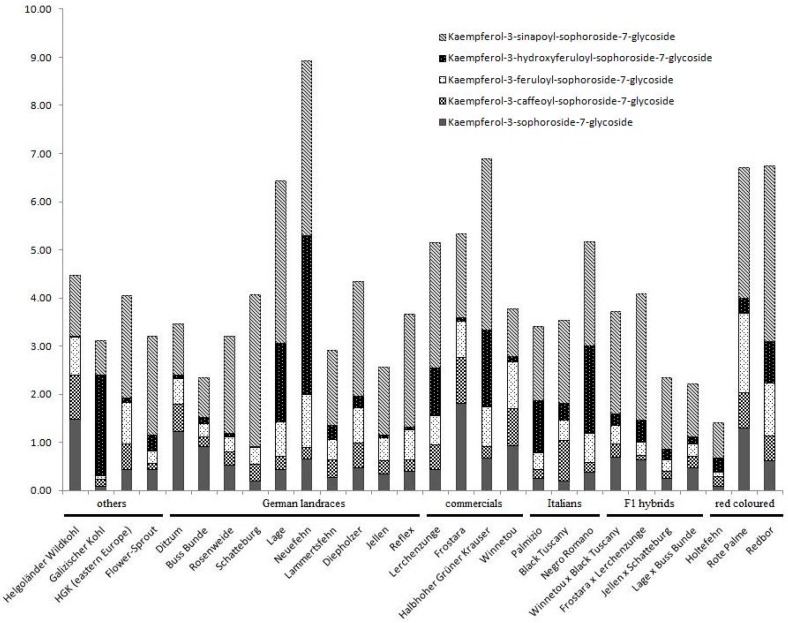
Kaempferol 7-glycoside contents quantified in kale cultivars (*Brassica oleracea* var. *sabellica*) via HPLC-MS analyses. Materials and methods are given in the Appendix.

The main flavonol glycosides of kale, as for other *Brassica oleracea* varieties, are non-acylated and acylated kaempferol glycosides [53,54,55]. Our intercultivar comparison supports high variability in these kaempferol glycosides, underlining the importance of investigating a large number of different cultivars before defining subgroup specific patterns (Figure 4). In all cultivars the monoacylated kaempferol-3-*O*-sinapoyl-sophoroside-7-*O*-glycoside was the main kaempferol glycoside, followed by either the monoacylated kaempferol-3-*O*-feruloyl-sophoroside-7-*O*-glycoside or non-acylated kaempferol-3-*O*-sophoroside-7-*O*-glycoside. However, some cultivars contained high concentrations of the monoacylated kaempferol-3-*O*-caffeoyl-sophoroside-7-*O*-glycoside (e.g., “Frostara” or “Winnetou”) and kaempferol-3-*O*-hydroxyferuloyl-sophoroside-7-*O*-glycoside (e.g., ”Lage”, “Neufehn”, “Lerchenzunge” or “Halbhoher grüner Krauser”). The acylated hydroxycinnamic acids of both compounds are characterized by a catechol structure that was shown to be important in kale’s response to UV-B [55]. Especially, the monoacylated kaempferol-3-*O*-caffeoyl-sophoroside-7-*O*-glycoside seems to be important for kale under high PAR and UVB radiation conditions [55,56]. However, based on on-line TEAC (Trolox Equivalent Antioxidant Capcity) data, kaempferol-3-*O*-caffeoyl-sophoroside-7-*O*-glycoside kaempferol-3-*O*-feruloyl-sophoroside-7-*O*-glycoside and kaempferol-3-*O*-hydroxyferuloyl-sophoroside-7-*O*-glycoside contribute equally to the antioxidant activity of kale [10].

Intercultivar differences in single and total anthocyanin contents were reported by numerous studies such as [57]. Our own study demonstrated that three derivative forms (cyanidin-3-(sinapoyl)-(sinapoyl)diglycoside-5-glycoside; cyanidin-3-(sinapoyl)-(feruloyl)diglycoside-5-diglycoside and cyanidin-3-(sinapoyl)(feruloyl)diglycoside-5-glycoside) were present exclusively in cultivars with red leaves or stems.

All these results support the assumption of great flavonoid variability within *B. oleracea* varieties, which complicates their differentiation based on flavonoids. Nevertheless, they also highlight the great potential of target-oriented breeding for flavonoid composition and content optimization. Thus, against our expectations we did not find any significant difference between specific cultivar subsets based on geography or morphological characteristics in case of flavonols, but anthocyanidins specific for red coloured accessions.

## 6. Future Perspectives

Due to the economic and scientific importance of *B. oleracea* and its nutritional value, comparisons are often made between edible plant parts under harvest conditions [30,45,49]. While such comparisons are understandable from a nutritional point of view, they are not useful for direct comparisons between *B. oleracea* varieties, because they blur varietal differences with those based on different harvest times or different plant tissues. Moreover, lots of parameters necessary for data reproduction or comparisons were often neglected or unmentioned, which impedes progress in flavonoid research. Another factor varying between previous studies is the choice of quantification method. Sophisticated techniques such as high-performance liquid chromatography (HPLC) are recommended here for further analyses on variety differentiation. A detailed overview on its handling, advantages and limits as well as suitable alternatives is given by Julkunen-Tiitto et al. [58]. This review also provides further information on sample handling and discusses current technical problems in flavonoid quantification.

Unfortunately, one issue cannot be solved by any quantification technique: the impossibility of quantifying total flavonoid contents. This is caused by the lack of available standards, the great number of different flavonoid compounds as well as the complexity of its derivative forms [58]. Measurements on a defined set of flavonoids are insufficient to make general statements on total flavonoid contents. Consequently, comparability can only be guaranteed given a clear and well defined set of substances. The choice of flavonoids and derivative forms naturally depends on the question of interest. Quantification and identification of anthocyanins have a great potential for cultivar differentiation [59] as well as variety identification and separation [47]. For example, our own investigations on kale included three glycosides, which were almost exclusively found in red coloured cultivars (see above). Furthermore, identification and quantification of flavonols quercetin and kaempferol are recommended for cultivar differentiation [11], variety identification and separation [8,15,60], as well as investigations on seasonal variation [3] and influence of cooking conditions [60]. Quantification of isorhamnetin is recommended for differentiation of varieties [15], as well as for investigations in cooking conditions [60]. In accordance with others, our results support cultivar specific variation in Q/K ratios and single flavonol glycoside contents [11]. Flavone quantification and identification are recommended in case of apigenin and luteolin, although it is yet unclear if they show any ontogenetic, seasonal or cultivar specific variation due to the sparse and contradicting remarks in the literature [29,43,44,45]. From our point of view, flavone quantification is of potential interest for variety as well as cultivar differentiation. Recommendations for standard selections in flavones are given by Lin et al. [54]. Finally, analyses on isoflavones such as daidzein and genistein are potentially useful for identification and separation of varieties [49,61]. As an example of more detailed investigations on isoflavones see Lapcik et al. [62], who used five isoflavone specific enzyme-linked immunosorbent assays (ELISAs) after HPLC sample fractionation to identify isoflavones in *Arabidopsis*.

To facilitate the selection of derivatives, we present a list of potential meaningful flavonoid glycosides for chemotaxonomic analyses on *B. oleracea* regarding anthocyanins as well as flavonols kaempferol and quercetin (see Table 3A,B). Future investigations may find other compounds equally suited for subgroup identification or reduce the list. However, there is promising evidence that these compounds are sufficiently variable to provide a means to distinguish between *B. oleracea* varieties. Furthermore, this set of flavonoids is suitable to guide in the choice of cultivars for target-oriented breeding and will improve future data comparability.

## Figures and Tables

**Table 1:** Selection of previous studies and the background information given in the corresponding publications.

Reference	*Brassica oleracea* Variety	Preharvest Conditions	Sample Material		Post-Harvest Conditions	Method	Total Flavonoid Content	Flavonoids Quantified
	Variety and Cultivar	Age; Nutrition, Light, Temperature	Plant Organ	Position & Age	Temperature & Storage Details		Considered as	
**Bahorun et al. [29]**	*italica* cv “Packman” *italica* cv “Kashmere” *capitata* cv “KKCross”	?	Flower Flower Leaves	?	Given in detail	HPLC	Quercetin equivalents	Myricetin, quercetin, kaempferol, apigenin and luteolin aglycones after acid hydrolysis
**Heimler et al. [30]**	*capitata* cv ?; *italica* cv ?; *acephala* cv ?; *sabauda* cv ?; *botrytis* cv “Verde di Macerata”, “Snow ball”, *gemnifera* cv “Zencher”	?	Edible part	?	Given in detail	HPLC	Catechin equivalents	Kaempferol-3-[2-sinapoylglucopyranosyl(1,2) glucopyranoside]-7-[glucopyranosyl(1,4) glucopyranoside], kaempferol-3-[-2-feruloylglucopyranosyl(1,2) glucopyranoside]-7-[glucopyranosyl(1,4) glucopyranoside], kaempferol tetraglycoside, kaempferol sinapoyl tetraglycoside, kaempferol cumaroyl tetraglycoside, kaempferol diglycoside; Quercetin-glucoside
**Jacob et al. [31]**	*capitata* cv?; *capitata* var. *rubra* cv?	?	Edible parts	?	?	Spectrophotometry, AlCl_3_	Epicatechin equivalents	Method is specific for rutin, luteolin and catechins (Pękal and Pyrzynska [26])
**Jaiswal et al. [32]**	*capitata* f. *alba* cv?; *italica* cv?; *gemmifera* cv?	?	Edible parts	Positions given, ages?	?	HPLC	Quercetin equivalents	?
**Lola-Luz et al. [33]**	*italica* cv “Ironman” and “Red Admiral”	Three-week range, Timepoints vary; Given in detail	Heads/florets	?	−20 °C, two-week range	Spectrophotometry, AlCl_3_	Catechin equivalents	Method is specific for rutin, luteolin and catechins (Pękal and Pyrzynska [26])
**Naguib et al. [23]**	*italica* cv “Calabrese”, “Southern star”	?; Given in detail; field cond.; location given	Florets	?	Given in detail	Spectrophotometry, AlCl_3_	Quercetin equivalents	Method is specific for rutin, luteolin and catechins (Pękal and Pyrzynska [26])
**Reilly et al. [12]**	*italica* cv “TZ6002”, “TZ5055”, “TZ5052”, “TZ4043”, “Red Admiral”, “Ironman”	Given in detail	Leaf, immature Primary floret, mature primary floret, secondary floret, flower	?	Given in detail	Spectrophotometry, AlCl_3_	Catechin equivalents	Method is specific for rutin, luteolin and catechins (Pękal and Pyrzynska [26])
**Valverde et al. [24]**	*italica* cv “Belstar”, “Fiesta”	Given in detail	Primary florets	?	−20 °C, 24-h range	Spectrophotometry, AlCl_3_	Catechin equivalents	Method is specific for rutin, luteolin and catechins (Pękal and Pyrzynska [26])

HPLC: High-performance liquid chromatography; ?: not specified; cond: conditions; cv: cultivar; AlCl_3_: aluminum chloride.

**Table 2:** Specific flavonoids commonly analysed in *Brassica oleracea* (function and intraspecific variation factor included).

Flavonoid (Subclass)	Physiological Function	Relevance in Medical Research	Content in *B. oleracea* [Method; Variety]	Intraspecific Variation Factor	References
Apigenin and luteolin (Flavone)	Nodulation and general defence mechanisms ^1^ Resistance to mycorrhization ^2^	Phytoestrogen with antibacterial and anti-inflammatory functions; apoptosis-inducer ^3^	Occurrence contradictory discussed ^4–6^; ~3-30 mg/kg fw [HPLC; *alba*, *botrytis*, *capitata*] ^7^	Apigenin ~2 Luteolin ~ 2.5 ^7^	^1^ Winkel-Shirley [40] ^2^ Ponce et al. [41] ^3^ Martens and Mithöfer [42] ^4−6^ Bahorum et al. [29]; Sakakibara et al. [43]; Miean and Mohamed [44] ^7^ Cao et al. [45]
Cyanidin (Anthocyanidin)	Pigmentation of flowers and fruits for recruitment of pollinators and seed dispersers ^1^	Antioxidant, anti-inflammatory, antimicrobial & anticarcinogenic activities, positive effect on visual performance & neuroprotection ^2^	23 cyanidin derivative forms [HPLC; *capitata* f *rubra*] ^3^; ~40–750 mg/kg fw [HPLC; *botrytis*; *capitata* f *rubra*] ^4^	18; qualitative dominance shift in derivative forms ^4^	^1^ Winkel-Shirley [40] ^2^ Erdman et al. [9] ^3^ Wu and Prior [46] ^4^ Scalzo et al. [47]
Daidzein, genistein, glycitein, biochanin A and formononetin (Isoflavone)	Root bacteria interaction including symbionts and pathogenic microorganism ^1^	Suggested to exert coronary benefits, directly reduce atherosclerosis and lower LDL-cholesterol ^2^	Max ~10 µg/100g fw [LC/MS/MS; *botrytis; capitata; capitata* f *alba; capitata* f *rubra; italica; gemmifera; sabellica; saubada*] ^3^	13 (for all listed isoflavones together) ^3^	^1^ Philips and Kapulnik [48] ^2^ Erdman et al. [9] ^3^ Kuhnle et al. [49]
Kaempferol (Flavonol)	Prevent oxidative stress in chloroplasts ^1^; ROS reduction ^1^; photoprotection ^1^; free radical scavenging capacity ^2^	Prevents coronary heart disease and chronic inflammation, suppresses cell proliferation in gut cancer lines, atherosclerosis prevention and growth inhibition of bacteria lines (gram-positive and gram-negative bacteria) ^2^	~60 mg/100 g fw [HPLC; *sabellica*] ^4^	Qualitative dominance shift in glycosides^2^; ontogenetic dependent variation with subgroup specific patterns^3^	^1^ Pollastri and Tattini [35] ^2^ Cartea et al. [8] ^3^ Soengas et al. [15] ^4^ Olsen et al. [50]
Quercetin (Flavonol)	See kaempferol; chelate transition metal ions, auxin gradient regulation^1^	See kaempferol	~45 mg/100 g fw [HPLC; *sabellica*] ^4^	Qualitative dominance shift in derivative forms ^2^; ontogenetic dependent variation with subgroup specific patterns ^3^	^1^ Pollastri and Tattini [35] ^2^ Cartea et al. [8] ^3^ Soengas et al. [15] ^4^ Olsen et al. [50]

Fw: fresh weight; LDL-cholesterol: low-density lipoprotein cholesterol; ROS: reactive oxygen species; LC/MS/MS: liquid chromatography tandem mass spectrometry; Superscripts in each row refer to the corresponding reference.

**Table 3:** List of potentially useful flavonoid derivatives for chemotaxonomic analyses on *Brassica oleracea*. A—Anthocyanins; B—Flavonols. Absence and presence partly suggested to be variety specific.

**A—Anthocyanins**	
**Anthocyanins (C—Cyanidin)**	***B. oleracea*** **Variety Verification**
C-3-(caffeoyl)-(*p*-coumaroyl)-diglycoside-5-glycoside	var. *capitata* f. *rubra* ^3^
C-3-(caffeoyl)-diglycoside-5-glycoside	var. *capitata* f. *rubra* ^3^
C-3-(caffeoyl)-diglycoside-5-glycoside	var. *capitata* f. *rubra* ^3^
C-3-(feruloyl)-(feruloyl)-diglycoside-5-glycoside	var. *capitata* f. *rubra* ^3^
C-3-(feruloyl)-diglycoside-5-glycoside	var. *botrytis itálica* ^1^ var. *capitata* f. *rubra* ^3^
C-3-(glycopyranosyl)-(feruloyl)-diglycoside-5-glycoside	var. *capitata* f. *rubra* ^3^
C-3-(glycopyranosyl)-(sinapoyl)-diglycoside-5-glycoside	var. *capitata* f. *rubra* ^3^
C-3-(*p*-coumaroyl)-(sinapoyl)-diglycoside-5-(malonyl)-glycoside	var. *botrytis italic* ^1^
C-3-(*p*-coumaroyl)-(sinapoyl)-diglycoside-5-glycoside	var. *botrytis itálica* ^1^ var. *capitata* f. *rubra* ^3^
C-3-(*p*-coumaroyl)-diglycoside-5-glycoside	var. *botrytis italic* ^1^
C-3-(*p*-hydroxybenzoyl)-(oxaloyl)-diglycoside-5-glycoside	var. *capitata* f. *rubra* ^3^
C-3-(sinapoyl)-(feruloyl)-diglycoside-5-(malonyl)-glycoside	var. *botrytis italic* ^1^
C-3-(sinapoyl)-(feruloyl)-diglycoside-5-glycoside	var. *botrytis itálica* ^1^ var. *capitata* f. *rubra* ^3^ var. *sabellica* ^4^
C-3-(sinapoyl)-(sinapoyl)-diglycoside-5-(malonyl)-glycoside	var. *botrytis italic* ^1^
C-3-(sinapoyl)-(sinapoyl)-diglycoside-5-glycoside	var. *botrytis itálica* ^1^ var. *capitata* f. *rubra* ^3^ var. *sabellica* ^4^
C-3-(sinapoyl)-diglycoside-5-(sinapoyl)-glycoside	var. *capitata* f. *rubra* ^3^
C-3-(sinapoyl)-diglycoside-5-glycoside	var. *botrytis itálica* ^1^ var. *capitata* f. *rubra* ^3^
C-3-(sinapoyl)-diglycoside-5-xyloside	var. *capitata* f. *rubra* ^3^
C-3-(sinapoyl)-glycoside-5-glycoside	var. *capitata* f. *rubra* ^3^
C-3-(sinapoyl)-triglycoside-5-glycoside	var. *botrytis italic* ^1^
C-3,5-diglycoside	var. *botrytis itálica* ^1^ var. *capitata* f. *rubra* ^3^
C-3-diglycoside	var. *capitata* f. *rubra* ^3^
C-3-diglycoside-5-glycoside	var. *botrytis itálica* ^1^ var. *capitata* f. *rubra* ^3^
C-3-diglycoside-5-xyloside	var. *capitata* f. *rubra* ^3^
C-3-(6-feruloyl)-sophoroside-5-(6-sinapyl)-glycoside	var. *botrytis^2^* and var. *capitata* ^2^
C-3-(6-feruloyl)-sophoroside-5-glycoside	var. *botrytis^2^* and var. *capitata* ^2^
C-3-(6-*p*-coumaryl)-sophoroside-5-(6-sinapyl)-glycoside	var. *botrytis^2^* and var. *capitata* ^2^
C-3-(6-*p*-coumaryl)-sophoroside-5-glycoside	var. *botrytis^2^* and var. *capitata* ^2^
C-3-(6-sinapyl)-sophoroside-5-(6-sinapyl)-glycoside	var. *botrytis^2^* and var. *capitata* ^2^
C-3-(6-sinapyl)-sophoroside-5-glycoside	var. *botrytis^2^* and var. *capitata* ^2^
C-3-glycoside-5-glycoside	var. *capitata ^2^*
C-3-sophoroside-5-glycoside	var. *botrytis* ^2^ and var. *capitata* ^2^
**B—Flavonols**	
**Flavonol (Q—Quercetin; K—Kaempferol)**	***B. oleracea*** **Variety Verification (as Reviewed by Cartea et al. [8])**
Q-3-*O*-sophorotrioside-7-*O*-sophoroside	var. *acephala*, var. *botrytis italica*
Q-3-*O*-sophorotrioside-7-glycoside	var. *acephala*, var. *botrytis italica*
Q-3-*O*-sophoroside-7-*O*-glycoside	var. *capitata* f. *alba*, var. *acephala*, var. *botrytis*, var. *botrytis italica*, var. *costata*
Q-3,7-di-*O*-glycoside	var. *capitata* f. *alba*, var. *acephala*, var. *botrytis italica*
Q-3-*O*-sophoroside	var. *capitata* f. *alba*, var. *acephala*, var. *botrytis italica*
Q-3-*O*-glycoside	var. *botrytis italica*, var. *costata*
Q-3-*O*-(caffeoyl)-sophorotrioside-7-*O*-glycoside	var. *botrytis italica*
Q-3-*O*-(sinapoyl)-sophorotrioside-7-*O*-glycoside	var. *botrytis italica*
Q-3-*O*-(feruloyl)-sophorotrioside-7-*O*-glycoside	var. *botrytis italica*
Q-3-*O*-(*p*-coumaroyl)-sophorotrioside-7-*O-*glycoside	var. *botrytis italica*
Q-3-*O*-(caffeoyl)-sophoroside-7-*O*-glycoside	var. *capitata* f. *alba*, var. *acephala*, var. *botrytis italica*
Q-3-*O*-(methoxycaffeoyl)-sophoroside-7-*O-*glycoside	var. *capitata* f. *alba*, var. *acephala*
Q-3-*O*-(sinapoyl)-sophoroside-7-*O*-glycoside	var. *capitata* f. *alba*, var. *acephala*, var. *botrytis*
Q-3-*O*-(*p*-coumaroyl)-sophoroside-7-*O*-glycoside	var. *botrytis italica*
Q-3-*O*-(feruloyl)-sophoroside	var. *capitata* f. *alba*, var. *acephala*, var. *botrytis italica*
K-3-*O*-tetraglycoside-7-*O*-sophoroside	var. *costata*
K-3-*O*-sophorotrioside-7-*O*-sophoroside	var. *capitata* f. *alba*, var. *acephala*, var. *botrytis*, var. *botrytis italica*, var. *costata*
K-3-*O*-sohorotrioside-7-*O*-glycoside	var. *botrytis*, var. *botrytis italica*, var. *costata*
K-3-*O*-sophoroside-7-*O*-diglycoside	var. *botrytis*, var. *botrytis italica*, var. *costata*
K-3-*O*-sophoroside-7-*O*-glycoside	var. *capitata* f. *alba*, var. *acephala*, var. *botrytis*, var. *botrytis italica*, var. *costata*
K-3,7-di-*O*-glycoside	var. *capitata f. alba*, var. *acephala*, var. *botrytis italica*
K-3-*O*-sophoroside	var. *capitata f. alba*, var. *acephala*, var. *botrytis italica*
K-7-*O*-glycoside	var. *capitata* f. *alba*, var. *acephala*, var. *botrytis*
K-3-*O*-glycoside	var. *botrytis italica*, var. *costata*
K-3-*O*-(caffeoyl)-sophorotrioside-7-*O*-sophoroside	var. *botrytis italica*
K-3-*O*-(methoxycaffeoyl)-sophorotrioside-7-*O*-sophoroside	var. *botrytis italica*
K-*O*-(sinapoyl)-sophorotrioside-7-*O*-sophoroside	var. *botrytis italica*
K-*O*-(feruloyl)-sophorotrioside-7-*O*-sophoroside	var. *botrytis italica*
K-3-*O*-(p-coumaroyl)-sophorotrioside-7-*O*-sophoroside	var. *botrytis italica*
K-3-*O*-(caffeoyl)-sophorotrioside-7-*O*-glycoside	var. *botrytis italica*
K-3-*O*-(methoxycaffeoyl)-sophorotrioside-7-*O-*glycoside	var. *botrytis italica*
K-*O*-(sinapoyl)-sophorotrioside-7-*O*-glycoside	var. *botrytis italica*
K-*O*-(feruloyl)-sophorotrioside-7-*O*-glycoside	var. *botrytis italica*
K-3-*O*-(caffeoyl)sophoroside-7-*O*-glycoside	var. *capitata* f. *alba*, var. *acephala*, var. *botrytis*, var. *botrytis italica*, var. *costata*
K-3-*O*-(methoxycaffeoyl)-sophoroside-7-*O*-glycoside	var. *capitata* f. *alba*, var. *acephala*, var. *costata*
K-3-*O*-(sinapoyl)-sophoroside-7-*O*-glycoside	var. *capitata* f. *alba*, var. *acephala*, var. *botrytis*, var. *costata*
K-3-O-(feruloyl)-sophoroside-7-*O*-glycoside	var. *capitata* f. *alba*, var. *acephala*, var. *botrytis*, var. *costata*
K-3-*O*-(p-coumaroyl)-sophoroside-7-*O*-glycoside	var. *capitata* f. *alba*, var. *acephala*
K-3-*O*-(methoxycaffeoyl)-sophoroside	var. *capitata* f. *alba*, var. *acephala*, var. *botrytis italica*
K-3-*O*-(sinapoyl)-sophoroside	var. *capitata* f. *alba*, var. *acephala*, var. *costata*
K-3-*O*-(feruloyl)-sophorotrioside	var. *costata*
K-3-*O*-(feruloyl)-sophoroside	var. *capitata* f. *alba*, var. *acephala*
K-3-*O*-(p-coumaroyl)-sophoroside	var. *capitata* f. *alba*, var. *acephala*

^1^ Moreno et al. [59]; ^2^ Scalzo et al. [47]; ^3^ Wu and Prior [46]; ^4^ Mageney et al. (unpublished; see Supplementary Material).

## Supplementary Materials

Supplementary materials can be accessed at: http://www.mdpi.com/1420-3049/22/2/252/s1. Table S1: Raw data of our own flavonoid quantifications on 28 kale (*Brassica oleracea* var. *sabellica*) varieties considering quercetin and kaempferol glycosides.

## Appendix A

### A1. Plant Material

Plants were grown under field conditions in the botanic garden, Carl-von Ossietzky-University Oldenburg, Northern Germany (53°9′ N, 8°13′ E) at Plaggen soil using a NPK + Mg fertilizer (12-8-16+3; 80 g/m^2^) plus stone powder (300 g/m^2^) directly before planted. Whole adult leaves of the second youngest rosette were harvested in December 2014 at noon, stored at −80 °C and freeze-dried. At the date of harvest, all selected plants were 38 weeks old.

### A2. Flavonol and Anthocyanin Quantification

Flavonoids were analyzed modifying the method of Schmidt et al. [11]. Lyophilized kale leafs (0.01 g) were extracted with 600 µL of 60% aqueous methanol on a magnetic stirrer plate for 40 min at 20 °C and centrifuged at 4500 rpm for 10 min at same temperature and the supernatant was collected in a reaction tube. Same process was repeated twice with 300 µL of 60% aqueous methanol for 20 min and 10 min respectively collecting the supernatant in the previous tube. The extract was subsequently evaporated till it was dry and suspended in 200 µL of 10% aqueous methanol. Again the solvent was centrifuged at 3000 rpm for 5 min at 20 °C and filtered through Corning CostarSpin-X plastic centrifuge tube filters (Sigma Aldrich Chemical Co., St. Louis, MO, USA) for the HPLC analysis. Each extraction was carried out in duplicate.

The flavonoids composition and concentrations were determined using a series 1100 HPLC (Agilent Technologies, Waldbronn, Germany) equipped with a degasser, binary pump, autosampler, column oven and photodiode array detector to determine the hydroxycinnamic acid derivatives and glycosides of flavonols. An Ascentis Express F5 column (150 mm × 4.6 mm, 5 µm, Supelco (Bellfonte, PA, USA)) was used to separate the compounds at 25 °C. Eluent A was 0.5% acetic acid and eluent B was 100% acetonitrile. Gradient used for eluent B was 5%–12% (0–3 min), 12%–25% (3–46 min), 25-90% (46–49.5 min), 90% isocratic (49.5–52 min), 90%–5% (52–52.7 min) and 5% isocratic (52.7–59 min). The determination was conducted at a flow rate of 0.85 mL·min^−1^ and wavelength of 330 nm and 370 nm for acylated flavonol glycosides and non-acylated flavonol glycosides, respectively. Glycosides of flavonols were identified as deprotonated molecular ions and characteristic mass fragment ions according to Schmidt et al. [11] by high-pressure liquid chromatograpy with a diode array detector coupled to an ion-trap mass spectrometer using electrospray ionization (HPLC-DAD-ESI-MS*^n^*) using an Agilent series 1100 ion trap mass spectrometer (Agilent, Waldbronn, Germany) in negative ionization mode. Nitrogen was used as the dry gas (10 L·min^−1^, 325 °C) in addition to nebulizer gas (40 psi) with a capillary voltage of −3500 V. Helium was used as the collision gas in the ion trap. The mass optimization for the ion optics of the mass spectrometer for quercetin was performed at *m/z* 301 or arbitrary at *m/z* 1000. The MS^n^ experiments were performed in auto up mode by HPLC-DAD-ESI-MS^3^ in a scan from *m/z* 200–2000. The standards, quercertin-3-glycoside, cyaniding-3-glycoside and kaempferol-3-glycoside (Roth, Karlsruhe, Germany) were used for external calibration curves. Results are given as mg·g^−1^ dry weight.
