# Supplementary material for: A Guide to the Variability of Flavonoids in Brassica oleracea
Source: Molecules. 2017 Feb 8;22(2):252. doi: 10.3390/molecules22020252 (PMC6155772; doi:10.3390/molecules22020252)
Supplement: Supplementary file 1 [file molecules-22-00252-s001.pdf]

Supplementary Materials: A Guide to the Variability of Flavonoids in *Brassica oleracea*

Vera Mageney, Susanne Neugart and Dirk C. Albach

Table S1. Raw data of own flavonoid quantifications on 28 kale (*Brassica oleracea* var. *sabellica*) accessions considering quercetin and kaempferol glycosides.

Quercetin-glc *1	Kaempferol-glc *2	Ratio [Q/K]	Accession	Description
8.61	7.41	1.16	Ditzum	German landraces
8.93	10.39	0.86	Buss Bunde	German landraces
4.29	3.09	1.39	Rosenweide	German landraces
4.36	5.54	0.79	Schatteburg	German landraces
2.96	4.82	0.61	Lage	German landraces
1.47	5.94	0.25	Neuefehn	German landraces
13.36	8.88	1.50	Lammertsfehn	German landraces
2.33	5.45	0.43	Diepholzer	German landraces
8.12	3.95	2.06	Jellen	German commercial
7.23	6.13	1.18	Reflex	German commercial
3.73	6.00	0.62	Halbhoher grüner Krauser	German commercial
0.60	7.66	0.08	Frostara	German commercial
8.14	8.84	0.92	Winnetou	German commercial
5.02	5.85	0.86	Lerchenzunge	German commercial
0.27	1.43	0.19	Black Tuscany	Italian varieties
0.24	2.13	0.11	Negro Romano	Italian varieties
1.20	5.52	0.22	Palmizio	Italian varieties
2.78	7.34	0.38	Winnetou x Black Tuscany	Hybrid lines
3.26	3.63	0.90	Frostara x Lerchenzunge	Hybrid lines
5.08	4.81	1.06	Jellen x Schatteburg	Hybrid lines
4.49	2.65	1.70	Lage x Buss Bunde	Hybrid lines
4.69	9.83	0.48	Holtefehn	Red coloured varieties
3.18	9.06	0.35	Rote Palme	Red coloured varieties
7.78	5.77	1.35	Redbor	Red coloured varieties
2.19	3.98	0.55	Helgoländer Wildkohl	Others
0.76	6.20	0.12	Galizischer Kohl	Others
5.84	4.23	1.38	Flower-Sprout	Others
0.47	3.01	0.16	HGK (eastern Europe)	Others

*1 comprising Quercetin-3-soph-7-glc, Quercetin-3-disin-triglc-7-glc, Quercetin-3-soph-7-sin-diglc, Quercetin-3-soph-7-fer-diglc, Quercetin-3-sin-soph-7-glc; *2comprising Kaempferol-3-soph-7-glc, Kaempferol-3-soph, Kaempferol-3-hfer-soph-7-diglc, Kaempferol-3-hfer-soph-7-glc, Kaempferol-3-caf-soph-7-glc, Kaempferol-3-sin-soph-7-diglc, Kaempferol-3-sin-soph-7-glc, Kaempferol-3-fer-soph-7-glc, Kaempferol-3-sin-soph, Kaempferol-3-fer-soph, Kaempferol-3-disin-triglc-7-glc; ratio [Q/K]: Quercetin/Kaempferol ratio; HGK: "Halbhoher grüner Krauser".
